# Intra-Oral Photograph Analysis for Gingivitis Screening in Orthodontic Patients

**DOI:** 10.3390/ijerph20043705

**Published:** 2023-02-19

**Authors:** Han-Na Kim, Kyuseok Kim, Youngjin Lee

**Affiliations:** 1 Department of Dental Hygiene, College of Health and Medical Sciences, Cheongju University, Cheongju 28503, Republic of Korea; 2 Department of Biomedical Engineering, Eulji University, Seongnam 13135, Republic of Korea; 3 Department of Radiological Science, College of Health Science, Gachon University, Incheon 21936, Republic of Korea

**Keywords:** gingivitis, intra-oral photo image, periodontitis, R/G value analysis, image processing framework for redness measurement

## Abstract

This study aimed to confirm the presence of gingival inflammation through image analysis of the papillary gingiva using intra-oral photographs (IOPs) before and after orthodontic treatment and to confirm the possibility of using gingival image analysis for gingivitis screening. Five hundred and eighty-eight (n  =  588) gingival sites from the IOPs of 98 patients were included. Twenty-five participants who had completed their orthodontic treatments and were aged between 20 and 37 were included. Six points on the papillary gingiva were selected in the maxillary and mandibular anterior incisors. The red/green (R/G) ratio values were obtained for the selected gingival images and the modified gingival index (GI) was compared. The change in the R/G values during the orthodontic treatment period appeared in the order of before orthodontic treatment (BO), mid-point of orthodontic treatment (MO), three-quarters of the way through orthodontic treatment (TO), and immediately after debonding (IDO), confirming that it was similar to the change in the GI. The R/G value of the gingiva in the image correlated with the GI. Therefore, it could be used as a major index for gingivitis diagnosis using images.

## 1. Introduction

The major dental diseases encountered in dentistry include dental caries, gingivitis, and periodontitis. Unfortunately, dentistry focuses on a downstream, patient-centered, curative, and rehabilitative approach to oral diseases [1]. Recently, there has been increased research on potential preventive approaches, with studies being conducted on the early diagnosis and screening of diseases before diagnosis [2]. Periodontal disease can be treated by dividing it into periodontitis and gingivitis. Gingivitis is a condition with no loss of alveolar bone in which the inflammation is limited to the gingiva; therefore, proper treatment can restore a healthy state [3]. Gingivitis is a reversible disease that can be restored to a healthy state, and it is necessary to prevent the disease from progressing to periodontitis through early diagnosis.

The diagnosis of gingivitis relies on the identification of signs and symptoms of inflammation resulting from the disease in gingival tissues. It is difficult for patients to recognize periodontitis independently, and patients often expect to recover from gingivitis with time rather than seeking to actively treat it. Invasive indices such as the gingival index (GI) [4] use a periodontal probe that is adapted to gently probe around the gingival sulcus of each tooth in the mouth to enable bleeding and to enable qualitative changes in the marginal and interproximal tissues to be recorded. It is important that patients recognize bleeding and red inflammation as signs of disease and visit a dental institution.

In addition to using a dental probe for gingivitis diagnosis, checking the depth of the periodontal pocket [5,6], and checking the level of inflammation through visual inspection, various methods have been attempted [7,8,9]. Many different methods have been introduced, and representative examples include diagnosis using saliva and diagnosis of diseases by X-ray using artificial intelligence technology [10,11]. These studies were performed to enable the diagnosis of diseases at an early stage so that treatment is possible and to enable diagnosis without visiting a dentist.

Recently, as economic conditions have improved and interest in aesthetics has increased, the number of patients undergoing orthodontic treatment has increased. Patients undergoing orthodontic treatment find it particularly difficult to maintain satisfactory oral hygiene because of the presence of bands, wires, and ligatures. In particular, after the removal of orthodontic braces, teeth may be in poor oral condition owing to advanced dental caries, gingival recession, and severe periodontitis [12,13,14,15]. Therefore, oral health education for orthodontic patients is emphasized, and dental staff need to relay its importance whenever patients visit for treatment. Orthodontic patients generally have intra-oral photographs (IOPs) taken before, during, and after orthodontic treatment to record the state of orthodontic treatment. In cases of gingivitis or periodontitis, the presence of red inflammation in the gingiva can be confirmed on intra-oral radiographs, and it is necessary to confirm the possibility of diagnosing oral diseases using intra-oral radiographs.

To date, there have been various attempts to diagnose diseases using IOPs, including oral cancer detection on an imaging system using a fluorescence enhancement method [16,17]. For the diagnosis of dental caries, an optical imaging system was used to distinguish sound teeth and caries lesions with quantitative values of the reflectance, transmittance, and absorbance [18].

Computer-assisted systems have been actively studied to improve the accuracy of dental health screening. In particular, radiographic and photographic intra-oral images have been intensively discussed when studying alveolar bone loss and periodontal disease [19,20,21]. Image analysis for periodontal diagnosis has mainly interpreted correlation with the disease by extracting features of abnormal symptoms, such as the degree of alveolar bone loss and periodontal redness. Sela et al. [22] introduced a method of structural analysis using morphological operators to segment trabeculae in dental X-ray images. This method has a limitation in that it is highly affected by degradation, such as noise. Statistical analysis based on methods including the gray level co-occurrence matrix (GLCM) [23], multichannel GLCM (MGLCM) [24], and domain transform methods [25] was applied to radiographic and photographic images to extract and classify features [26]. Based on the results of the extracted features, gingivitis, periodontitis, and the stage of periodontal disease were predicted. However, it is difficult to present a linear relationship between the extracted features and disease; therefore, there is a limit to deriving highly accurate results.

Recently, machine learning-based approaches have shown superior results in the diagnosis of periodontitis [27,28]. Periodontal disease prediction methods based on convolution neural networks predict diseases with high accuracy using multiple radiographs, optical photographs, and patient information [29,30,31]. Chang et al. [32] demonstrated that high accuracy and reliability were achieved by training each network suitable for the purpose and providing the derived results by synthesizing them into one panoramic image. Li et al. [33,34] attempted to diagnose and predict gingivitis using a particle swarm optimization neural network incorporating contrast-limited adaptive histogram equalization and MGLCM methods. This combination approach derived more accurate and sensitive results compared to state-of-the-art methods. However, data-driven prediction methods are dependent on the data size and acquisition conditions. Therefore, it is necessary to verify the reference data (or label data) with considerable expertise and time.

A study that attempted to screen for gingivitis automatically using deep learning technology suggested that the area under the curve for detecting gingivitis, dental calculus, and soft deposits was 87.11%, 80.11%, and 78.57% using oral photos [31]; however, since the range was wide and oral sites not related to gingival inflammation were included, the reliability of their findings between the actual inflammation occurrence and the target data need to be confirmed. This study aimed to confirm the presence or absence of inflammation through image analysis of the papillary gingiva using IOPs before and after orthodontic treatment and to confirm the possibility of using the gingival image analysis method for periodontal disease screening.

## 2. Materials and Methods

### 2.1. Study Participants

Ninety-eight oral photos, including 588 targeted gingivae, were captured from 25 patients admitted at three orthodontic dental clinics in Cheongju city, South Korea, between September 2018 and November 2022. A power analysis was performed using the G*Power software, version 3.1.9.2 (Heinrich-Heine-Universität Düsseldorf, Düsseldorf, Germany), using mean differences, with mean differences of 0.7% for gingivitis from a related study. An error probability of 0.05 and actual power of 0.95 were used. The number of subjects calculated through the G*Power software was 23. The number of subjects initially included in the study was 30, but in the process of evaluating the quality of the obtained photographs the final number of subjects was selected as 25 participants.

A dental hygienist captured all the photos. The patients were aged between 20 and 39 years. Gingivitis was considered the primary disease in this study. The inclusion criteria were those who had completed orthodontic treatment in their 20s and 30s, had undergone orthodontic treatment for at least six months, had received orthodontic treatment with fixed orthodontic devices, and had been diagnosed with gingivitis by a dentist during orthodontic treatment.

Patients were excluded from the study if they were aged 40 years or older, had systemic disease (such as diabetes and hypertension), had dental caries on the buccal smooth tooth surface, had undergone tooth extraction during orthodontic treatment, had noticeable discoloration of their teeth or had severe melanin pigmentation on the gingiva, or had gingiva that was dark red even though there was no inflammation. In addition, patients with abnormal anatomical structures due to periodontal disease were excluded. The details of exclusion criteria were as follows: subjects with a systemic disease within the past 6 months who are receiving continuous treatment, subjects with other diseases that cause inflammatory transformation of the gingiva, including oral cancer, or subjects taking antibiotics due to disease. Among patients aged ≥ 40 years, some patients with advanced periodontitis were included. Advanced periodontitis can cause alveolar bone loss and gingival recession. The size of the gingiva can be different depending on the participants’ oral condition due to the deformation of the gingiva; therefore, the size of the gingiva was not considered. None of the patients in this study had dental caries on smooth surfaces.

The IOPs used in the research analysis were received by one data organizer who attached a number to each photograph to prevent exposure to personal information and ensure the patients’ anonymity. Then the IOPs were prepared for analysis. The requirement for informed consent was waived because this was a retrospective study and all data were anonymized. We received an exemption for institutional review board review from the Bioethics Review Committee of Cheongju University (1041107-202212-HR-053-01).

### 2.2. Targeted Gingiva

**Figure 1:**
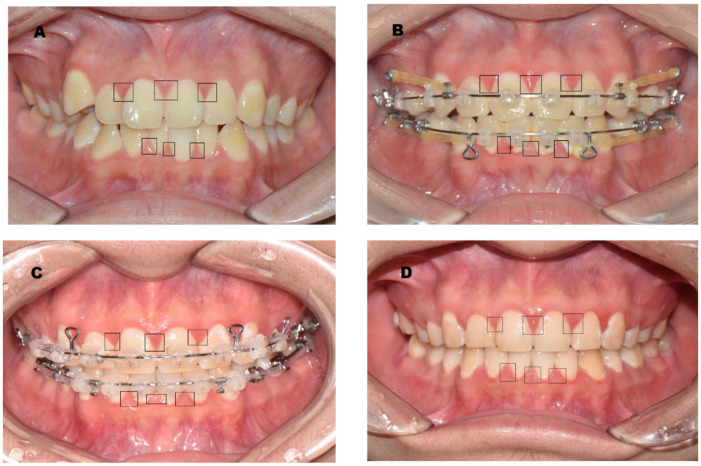
IOP showing the target gingiva: The lesions in the black squares in the IOP were papillary gingiva, which were selected and analyzed to measure redness. ((**A**), BO, before orthodontic treatment; (**B**), MO, mid-point of orthodontic treatment; (**C**), TO, three-quarters of the way through orthodontic treatment; (**D**), IDO, immediately after debonding).

A total of 588 targeted gingiva samples were included. The gingiva selected included six points of papillary gingiva in the maxillary and mandibular anterior incisors (FDI dental numbering system: Nos. 13, 12, 11, 21, 22, 23, 43, 42, 41, 31, 32, and 32) from 25 participants. As the orthodontic treatment progressed, four IOPs were taken before orthodontic treatment (BO, see the Figure 1A), mid-point of orthodontic treatment (BO, see the Figure 1B), three-quarters of the way through orthodontic treatment (TO, see the Figure 1C), and immediately after debonding (IDO, see the Figure 1D).

### 2.3. Proposed Framework to Measure the Redness

**Figure 2:**
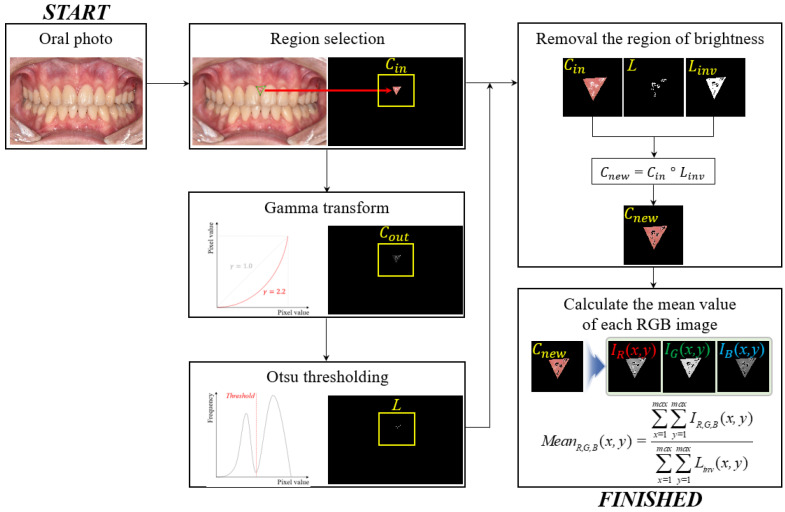
Simplified illustration of the proposed redness measurement framework using a standard oral photograph.

Figure 2 shows a simplified flowchart of the proposed redness measurement scheme, which involves extracting several areas on the upper and lower gums around the tooth using high-definition imaging. First, oral images are acquired by a standard protocol using an advanced 4 k ultra high-definition (4 k UHD) optical camera (Nikon, CMOS-type, 24.2 Megapixels, AF-S NIKKOR 85 MM F1.4 G telephoto lens), and then the region is selected to measure the redness. The selected region, $C_{in}$, is composed of a three-dimensional image (i.e., width, height, and depth), which can be expressed as follows:

(1) $$C_{in}=\{\begin{array}{c} C_{R}\left(x,y\right)\\ C_{G}\left(x,y\right)\\ C_{B}\left(x,y\right) \end{array}\;\;0\leq C_{R,G,B}\left(x,y\right)\leq 255,$$

where x and y denote the coordinates of the oral image. Here, when the flash is used to ensure the appropriate brightness of the image, the halo artifact is often included in the periodontal region to obtain an image. We performed gamma correction [35] to emphasize the high intensity of the halo artifacts and the traditional gamma transformation given by

(2) $$C_{out}=bC_{in}{}^{\gamma }$$

where $C_{out}$ is the result of the gamma correction, which is obtained by applying the two constant parameters of b and γ to control the shape of the transformation curve. In this study, we used b=1.0 and γ=2.2, empirically. The corresponding values are not fixed and can change according to the exposure conditions in oral photography. The region of the artifact generated by the flash was separated using the Otsu method [36]. L is an index map with halo artifacts and $L_{inv}$ is the opposite. $C_{new}$, which is an artifact-removed image, is generated by the element product between $C_{in}$ and $L_{inv}$. Here, ∘ is an element-wise multiplication operator. Finally, the mean values of the three-color channels, $Mean_{R,G,B}\left(x,y\right)$, were calculated as follows:

(3) $$Mean_{R,G,B}\left(x,y\right)=\frac{\sum_{x=1}^{max}\sum_{y=1}^{max}I_{R,G,B}\left(x,y\right)}{\sum_{x=1}^{max}\sum_{y=1}^{max}L_{inv}\left(x,y\right)}$$

where $I_{R,G,B}\left(x,y\right)$ indicate the intensity values of the three-color channels in $C_{new}\left(x,y\right)$. These processes were repeated in other areas, and datasets for statistical analysis were collected.

Based on the above descriptions, we implemented the proposed algorithm using MATLAB ^TM^ version 8.3 (MathWorks, MA, USA) programming language and a normal workstation (operating system: Windows 10, CPU: 2.13 GHz, RAM: 64 GB). The obtained image was in JPEG format, and the image dimensions were 3300 × 5000 × 3.

### 2.4. Modified Gingival Index (GI)

This study attempted to confirm the association between redness obtained from the images and the GI. The degree of gingivitis, which can be confirmed by visual inspection, was scored using a modified GI [37]. The degree of gingival inflammation, which reflected the same lesion as the red/green (R/G) ratio analysis of the participants in the IOP, was scored as 0–4 points with the modified GI according to the criteria presented by Tobias et al. (Table 1) [37]. One researcher (HK), a dental hygienist, performed the GI analysis. The degree of gingivitis was confirmed by visually checking the images on the same computer. Duplicate analysis of 10% of all subject photos confirmed that the concordance of the results was more than 95%.

### 2.5. Statistical Methods

A descriptive statistical analysis of the gingival R/G ratio values at four time points during orthodontic treatment was performed. As a descriptive statistical analysis, the average and standard deviation of the gingival R values were presented for each of the six regions, and a correlation analysis between the modified GI and the highest R/G value was performed using the maxilla and mandible using Spearman’s test. According to orthodontic treatment, the differences in the R/G values were confirmed using Friedman’s and Kendall’s W. All analyses were performed using SPSS version 24.0 (IBM Corp., New York, NY, USA), and the alpha levels were set at 0.05. A value of 0.1 or less as a significance level was set as a tendency.

## 3. Results

### 3.1. General Characteristics

Twenty-five patients underwent orthodontic treatment. The study included eleven males and fourteen females. Of the participants, 75.7% and 24.3% were in their 20s and 30s, respectively. The average duration of orthodontic treatment was 22.1 months. The orthodontic devices used by the study subjects were mostly ceramic orthodontic devices (64%) (Table 2).

### 3.2. Gingival Index (GI)

Table 3 shows the changes in the GI according to the progress of orthodontic treatment. Although there was a difference in the degree according to the six gingival locations, gingival inflammation confirmed by the GI was confirmed to increase IDO and TO. In contrast, there was less inflammation BO and TO (*p* < 0.05).

### 3.3. R/G Ratio

Table 4 shows changes in the R/G ratio according to the orthodontic treatment. Changes in the R/G values in the IOPs according to the progress of orthodontic treatment were confirmed in each of the six gingivae. The results of the R/G values analysis confirmed that IDO, TO, and MO were high in that order (*p* < 0.05).

### 3.4. Correlation between the GI and R/G Values

Table 5 shows the results of the correlation analysis between the GI and R/G values. The correlation between the degree of gingival inflammation was visually confirmed, and the degree of redness was confirmed using photographic image analysis. The correlation coefficients in the maxilla were 0.63 for GI_MO and R/G_MO, 0.70 for GI_TO and R/G_TO, and 0.87 for GI_IDO and R/G_IDO, respectively (*p* < 0.05). Correlation coefficients in the mandible were 0.60 for GI_TO and R/G_TO and 0.73 for GI_IDO and R/G_IDO, respectively. (*p* < 0.05).

## 4. Discussion

Inspection of conditions such as color and contour changes in the gingiva is important in periodontal clinical examinations and disease determination [38,39]. It is important to confirm changes in the gingiva at a preclinical stage during the course of the disease. Since gingivitis is a stage without loss of the alveolar bone, it must be detected through early screening and appropriate measures should be taken. In this study, we compared the GI confirmed by visual inspection with the R/G value in the actual image to check whether the result of the image analysis was similar to the image and confirm the degree of gingival inflammation during orthodontic treatment. In this study, by analyzing photographs of suspected gingivitis using a recently developed image analysis method, it was found that the R/G value of the gingiva in the image can be used as an important indicator for gingivitis diagnosis.

Previous studies using images have attempted to confirm periodontal inflammation. However, the R/G ratio was confirmed in a state that included part of the teeth or buccal mucosa beyond the gingival region in the image [33,34,35]. As a result, the method for selecting the target lesion was reconsidered to ensure reliability. In this study, we attempted to improve the precision of the research results by sectioning and analyzing only the gingiva papillary [40,41], where gingival inflammation first begins. Additionally, the results obtained from the images were compared with the GI, a clinical indicator, to confirm the reliability of the actual data, thereby increasing the value of the study.

The inflammation of the gingiva confirmed by the GI increased immediately after debonding and three-quarters of the way through orthodontic treatment. In contrast, less inflammation was apparent before orthodontic treatment, at the mid-point of orthodontic treatment, and at the beginning of orthodontic treatment with orthodontic appliances. These findings confirm that there is an increase in gingivitis due to poor oral hygiene management with braces. Relatively little inflammation was observed at the mid-point of orthodontic treatment. However, patients received oral hygiene management education at the beginning of orthodontic treatment, and it was confirmed that the degree of inflammation was the lowest among those with a high interest in oral health. Ozlu et al. [42] reported that the Loë–Silness index is frequently used and that the second GI is often used as a secondary outcome to confirm the effectiveness of oral education in patients undergoing orthodontic treatment. However, the GI varies depending on the general condition of patients and the targeting area of the GI does not include the bonded dental surfaces. Bardal et al. emphasized [43] that oral healthcare guidance should be provided before and during treatment. Previous studies have suggested the use of mobile applications to deliver oral hygiene information and increase the efficiency of education [44,45].

During periodontal disease, host inflammatory cells are recruited and inflammatory cytokines such as IL-1β, IL-6, and TNF-α are released from fibroblasts, macrophages, connective tissue, and junctional epithelial cells. In particular, prostaglandins appear and increase microvascular permeability, and the color of the gingiva turns red or dark red [45]. The change in the R/G value during the orthodontic treatment period appeared in the order of immediately after the removal of the orthodontic appliance, three-quarters of the way through the orthodontic treatment, before the orthodontic appliance, and at the beginning of the orthodontic treatment, confirming that it was similar to the change in the GI. In other words, it was confirmed that the R/G value had a similar tendency to the change in the ranking of the GI value. Jönsson et al. [46] demonstrated the effectiveness of an individually tailored oral health education program for oral hygiene self-care in patients with chronic periodontitis.

The validation of the GI was significantly correlated with histological parameters of inflammation during gingivitis development; specifically, the infiltrated connective tissue volume and its ratio with the volume of non-infiltrated connective tissue increased with increasing GI [47]. In the results of the correlation analysis between the R/G and GI, the highest value of each R/G and GI was selected and analyzed in the upper and lower jaws of the three gingivae. The correlation coefficient between the R/G and GI was not confirmed in the gingiva before orthodontic treatment. However, a coefficient of 0.63–0.87 was confirmed three-quarters of the way through orthodontic treatment and immediately after debonding. These results show that the R/G value has limitations in reflecting the degree of gingival inflammation in a state of low gingival inflammation, but the correlation was confirmed with GI scores of two or three where gingivitis occurred as a result of orthodontic treatment. However, in the image analysis, the gingival volume or edema due to inflammation was not reflected, and only the color change and R/G ratio were obtained. Therefore, some of the variables were not significantly confirmed in the results of the correlation analysis.

The proposed method can derive the correlation between gingivitis and periodontitis from oral images without additional devices. However, several limitations need to be addressed to improve the performance. First, it is difficult to maintain the dynamic range under various exposure conditions, and it is necessary to unify the dynamic range of the obtained oral images by using the absolute intensity of the RGB color image. This problem is currently being studied through research and development. Another limitation is the accurate segmentation of the area saturated by the camera flash. The saturated value influences the result, and this problem can be overcome using more complicated methods, including the energy minimization method [48], Gaussian mixture model [49], prior knowledge-based method [50,51], multiscale-based method [52], and random walk method [53]. However, these approaches increase the computation time. Recently, deep-learning-based image segmentation methods have shown promising results [54] and are expected to improve the usefulness of the proposed method.

Another limitation of the current study is that we could not include patients over 40 years old because of the possibility of gingival recession in the oral cavity. In patients over 40 years old, the shape of the papillary gingiva on imaging is different for each patient, which could cause a difference in the results. Standardized IOPs using digital cameras, excluding personal cell phones, were used for the analysis, so many photos could not be included in the current study. Future studies should be conducted that include a larger number of samples. The anatomical structure in the oral cavity is three-dimensional, but images are two-dimensional, so gingival edema or morphological changes were not considered in the current study. For this, it is necessary to check whether the volume and shape have changed in a future study using IOPs while expanding the age range of the study subjects. The failure to secure a sufficient number of study subjects is considered to be a limitation of this study. However, since the number of analyzed gingival regions was sufficient, significant results were confirmed during statistical analysis. A study that secures a sufficient number of research subjects is planned for the future 

Nevertheless, our findings are still valuable. The current study shows that it is important to compare and judge gingival inflammation using images and the GI within the range designated by experts. Furthermore, since the correlation between the two indicators has been confirmed, the analysis method using images can be used.

Due to poor oral hygiene management, periodontal disease is accelerated by biofilms and bacteria in interdental spaces or oral environments. The relationship between cardiovascular disease and diabetes has been reported in many studies [55,56]. Moreover, increased carious teeth can be positively associated with the risk of cerebral or myocardial infarction [57]. As oral health management is a major factor in overall health, promoting oral hygiene and reducing oral inflammation must be continuously highlighted to patients. If an image analysis related to periodontal inflammation screening can be developed, patients’ overall health could be greatly improved.

## 5. Conclusions

In this study, a recently developed image analysis method was applied to analyze gingivitis using IOPs of orthodontic patients. It was confirmed that the R/G value of the gingiva in the image was correlated with the GI. The change in the R/G value during the orthodontic treatment period appeared in the order of immediately after the removal of the orthodontic appliance, three-quarters of the way through orthodontic treatment, before the orthodontic appliance, and at the beginning of the orthodontic treatment, confirming that it was similar to the change in the GI. In addition, the change in the degree of gingivitis according to the progress of orthodontic treatment was confirmed using the R/G values, showing that it could be used as a major index for gingivitis diagnosis using images. 

## Figures and Tables

**Table 1:** Modified gingival index scoring system [37].

Score	Diagnosis	Modified Gingival Index Criteria	Color	Texture	Volume	Extent
0	Healthy	Absence of inflammation	Normal	Normal	Normal	None
1	Mild inflammation (partial unit)	Slight change in color, a little change in the texture of any portion of, but not the entire, marginal or papillary gingival unit	Slightly more reddish or bluish-reddish	Slightly glazy	Slight edema of the margin	Part of the gingival unit
2	Mild inflammation (entire unit)	Criteria as above but involving the entire marginal or papillary gingival unit	Slightly more reddish or bluish-reddish	Slightly glazy	Slight edema of the margin	Entire gingival unit
3	Moderate inflammation	Glazing, redness, edema, and/or hypertrophy of the marginal or papillary gingival unit	Red or reddish-blue	Glazy	Edema and /or hypertrophy of the margin	Entire gingival unit
4	Severe inflammation	Marked redness, edema, and/or hypertrophy of the marginal or papillary gingival unit, spontaneous bleeding congestion, or ulceration	Markedly red or reddish-blue	Spontaneous bleeding or ulceration	Edema and/or hypertrophy of the entire unit	Entire gingival unit

**Table 2:** General characteristics of the participants.

Characteristics	Items	N (%)
Sex	Male	11 (44.0)
	Female	14 (56.0)
Age	20s	19 (75.7)
	30s	6 (24.3)
Average period of orthodontic treatment (Unit: Month, mean ± SD)	22.1 ± 13.0	
Orthodontic devices	Metal bracket	9 (36.0)
	Ceramic bracket	16 (64.0)

**Table 3:** GI of papillary gingivitis in the IOPs.

Orthodontic Treatment	Tooth Region			Tooth Region		
	#12-#13	#11-#21	#22-#23	#43-#43	#41-#31	#32-#33
BO	0.80 ± 0.71	0.57 ± 0.65	0.50 ± 0.76	0.57 ± 0.85	0.64 ± 1.01	0.54 ± 0.97
MO	0.82 ± 0.85	0.71 ± 0.91	0.64 ± 0.80	0.93 ± 0.83	1.00 ± 0.88	0.92 ± 0.86
TO	1.50 ± 1.09	1.50 ± 0.85	1.57 ± 0.94	1.79 ± 1.12	1.71 ± 0.91	1.92 ± 1.12
IDO	1.64 ± 0.86	1.79 ± 1.05	1.64 ± 0.77	1.86 ± 0.86	2.07 ± 0.92	1.85 ± 0.99
*p*-value *	0.002	0.001	0.001	<0.001	0.001	0.001

Mean ± SD. * Friedman test, Kendall’s W test. BO, before orthodontic treatment; MO, mid-point of orthodontic treatment; TO, three-quarters of the way through orthodontic treatment; IDO, immediately after debonding.

**Table 4:** R/G ratio of papillary gingivitis in the IOPs.

Orthodontic Treatment	Tooth Region			Tooth Region		
	#12-#13	#11-#21	#22-#23	#43-#43	#41-#31	#32-#33
BO	1.76 ± 0.26	1.58 ± 0.15	1.83 ± 0.23	1.59 ± 0.16	1.54 ± 0.12	1.58 ± 0.17
MO	1.59 ± 0.20	1.48 ± 0.15	1.66 ± 0.20	1.51 ± 0.15	1.48 ± 0.13	1.51 ± 0.15
TO	1.71 ± 0.21	1.57 ± 0.13	1.79 ± 0.18	1.59 ± 0.13	1.59 ± 0.11	1.63 ± 0.15
IDO	1.81 ± 0.29	1.66 ± 0.20	1.88 ± 0.22	1.71 ± 0.16	1.67 ± 0.14	1.72 ± 0.17
*p*-value *	<0.001	<0.001	0.001	<0.001	<0.001	<0.001

Mean ± SD. * Friedman test, Kendall’s W test. BO, before orthodontic treatment; MO, mid-point of orthodontic treatment; TO, three-quarters of the way through orthodontic treatment; IDO, immediately after debonding.

**Table 5:** Correlation analysis between the highest GI and R/G values of each maxillary and mandibular gingiva.

Location	Orthodontic Treatment	Maxilla				Mandible			
		R/G_BO	R/G_MO	R/G_ TO	R/G_ IDO	R/G_BO	R/G_MO	R/G_ TO	R/G_ IDO
Maxilla	GI_BO	0.43							
	GI_MO		0.63 *						
	GI_TO			0.70 **					
	GI_IDO				0.87 **				
Mandible	GI_BO					0.41			
	GI_MO						0.40		
	GI_TO							0.60 *	
	GI_IDO								0.73 **

* *p* < 0.01, ** *p* < 0.05. Spearman’s Rho test with each of the values was the highest among the three papillary gingivae in the maxillary and mandible. GI, gingival index; R/G, red/green ratio; BO, before orthodontic treatment; MO, mid-point of orthodontic treatment; TO, three-quarters of the way through orthodontic treatment; IDO, immediately after debonding.

## Data Availability

Not applicable.
